# The clinical utility of combining D-dimer with the C-reactive protein/albumin ratio for assessing treatment response in pediatric infectious mononucleosis

**DOI:** 10.3389/fmed.2026.1830883

**Published:** 2026-05-15

**Authors:** Long Ying Zhu, Liang Zhang, Shui Fu, Zuo Jie Li

**Affiliations:** 1 Department of Clinical Laboratory, First People's Hospital of Linping District, Hangzhou, Zhejiang Province, China; 2 School of Medical Technology and Information Engineering, Zhejiang Chinese Medical University, Hangzhou, Zhejiang Province, China; 3 Department of Clinical Laboratory, The People's Hospital of Cangnan Zhejiang, Wenzhou, Zhejiang Province, China

**Keywords:** atypical lymphocytes percentage (ALC), C-reactive protein-to-albumin ratio, D-dimer, infectious mononucleosis, risk stratification, short-term changes

## Abstract

**Objective:**

This study evaluates the effectiveness of tracking D-dimer levels and the CRP/ALB ratio for objective quantification of therapeutic response and complication risk in children with infectious mononucleosis (IM).

**Methods:**

This study involved 171 hospitalized children diagnosed with IM, comprising 116 patients without complications and 55 with complications. Measurements of D-dimer, CRP, albumin, and atypical lymphocytes percentage (ALC) were taken within 24 h of admission and again on the fifth day of treatment to calculate the rate of change. Independent predictors were identified through logistic regression analysis, and the model's performance was validated using receiver operating characteristic (ROC) curves in both training and validation cohorts.

**Results:**

Although baseline markers were comparable between groups, post-treatment analysis revealed that the complications group exhibited persistent hypercoagulability and inflammation, as evidenced by elevated levels of D-dimer and CRP/ALB, along with a slower decline in atypical lymphocytes percentage (ALC) (*P* < 0.05). In contrast, the no-complication group demonstrated significantly greater reductions in ΔD-dimer, ΔCRP, ΔALC, and ΔCRP/ALB compared to the complication group. Multivariable analysis identified fever duration, ΔALC, ΔD-dimer and ΔCRP/ALB as independent predictors of complications. The composite model achieved an area under the curve (AUC) of 0.91 (95% CI 0.86–0.96), with a sensitivity of 0.85 and a specificity of 0.86. The model demonstrated strong performance in the validation cohort, achieving an AUC of 0.93, along with good calibration.

**Conclusions:**

Short-term changes of routine markers, particularly D-dimer and CRP/ALB, in conjunction with fever duration, may facilitate the objective evaluation of therapeutic efficacy and assist in risk stratification for complications in pediatric IM.

## 1 Introduction

Infectious mononucleosis (IM) represents an acute lymphoproliferative disorder resulting from a primary infection with the Epstein–Barr virus (EBV) (1). EBV, a ubiquitous γ-herpesvirus, infects over 95% of the global population. While the majority of EBV infections remain asymptomatic, IM frequently manifests clinically in adolescents and young adults (2). The characteristic clinical features of IM encompass fever, pharyngitis, and lymphadenopathy; nonetheless, severe complications, including splenic rupture, myocarditis, and meningitis, can arise in certain patients (3). The evaluation of therapeutic efficacy in IM has historically presented significant challenges. Traditional methodologies predominantly depend on clinical observation or the monitoring of Epstein-Barr virus (EBV) DNA levels as a singular parameter. While the assessment of EBV DNA load is crucial in evaluating the disease, relying solely on this metric may not suffice for a thorough appraisal of a patient's condition. A multidimensional assessment that synthesizes clinical findings with EBV DNA load and additional biomarkers could offer a more comprehensive and precise framework for the diagnosis and management of IM (4–6). This integrated approach has the potential to enhance diagnostic accuracy and facilitate the development of individualized treatment regimens.

In patients with IM, early alterations in inflammatory responses, coagulation function, and nutritional status are commonly observed and serve as indicators of both disease activity and therapeutic efficacy (5, 7). Consequently, assessing the “inflammation–coagulation–nutrition” axis is essential for identifying early treatment responses. Recent studies have explored the use of individual biomarkers, such as C-reactive protein (CRP), albumin (ALB), and D-dimer, to monitor disease progression. However, these biomarkers generally reflect only a single aspect of the underlying pathophysiology (5, 8). In contrast, D-dimer, CRP, and ALB measurements are already part of routine hematological testing in hospitalized patients, offering the benefits of no additional sampling, low testing costs, and rapid turnaround times (9). If the dynamic trajectories of these routinely collected markers can be effectively integrated into a multidimensional tool for assessing treatment response, there is significant potential to enhance risk stratification and individualized therapeutic management in IM (10).

Dynamic alterations in hematologic inflammatory and coagulation indices can effectively capture the host's response to therapy and reflect the evolving multisystem pathophysiological status (11). While routine hematologic testing provides absolute measurements of D-dimer, CRP, and ALB, the utility of static, single-time-point values for evaluating treatment response has yet to be systematically elucidated (10). Through the analysis of longitudinal trajectories derived from repeated in-hospital measurements, we have proposed a preliminary multiparametric evaluation model to assess therapeutic efficacy in infectious mononucleosis. This system is designed to quantify the temporal evolution of the “inflammation-coagulation-nutrition” axis, potentially providing an objective indicator of the clinical trajectory to complement bedside assessment.

## 2 Materials and methods

### 2.1 Study design and study population

**Figure 1:**
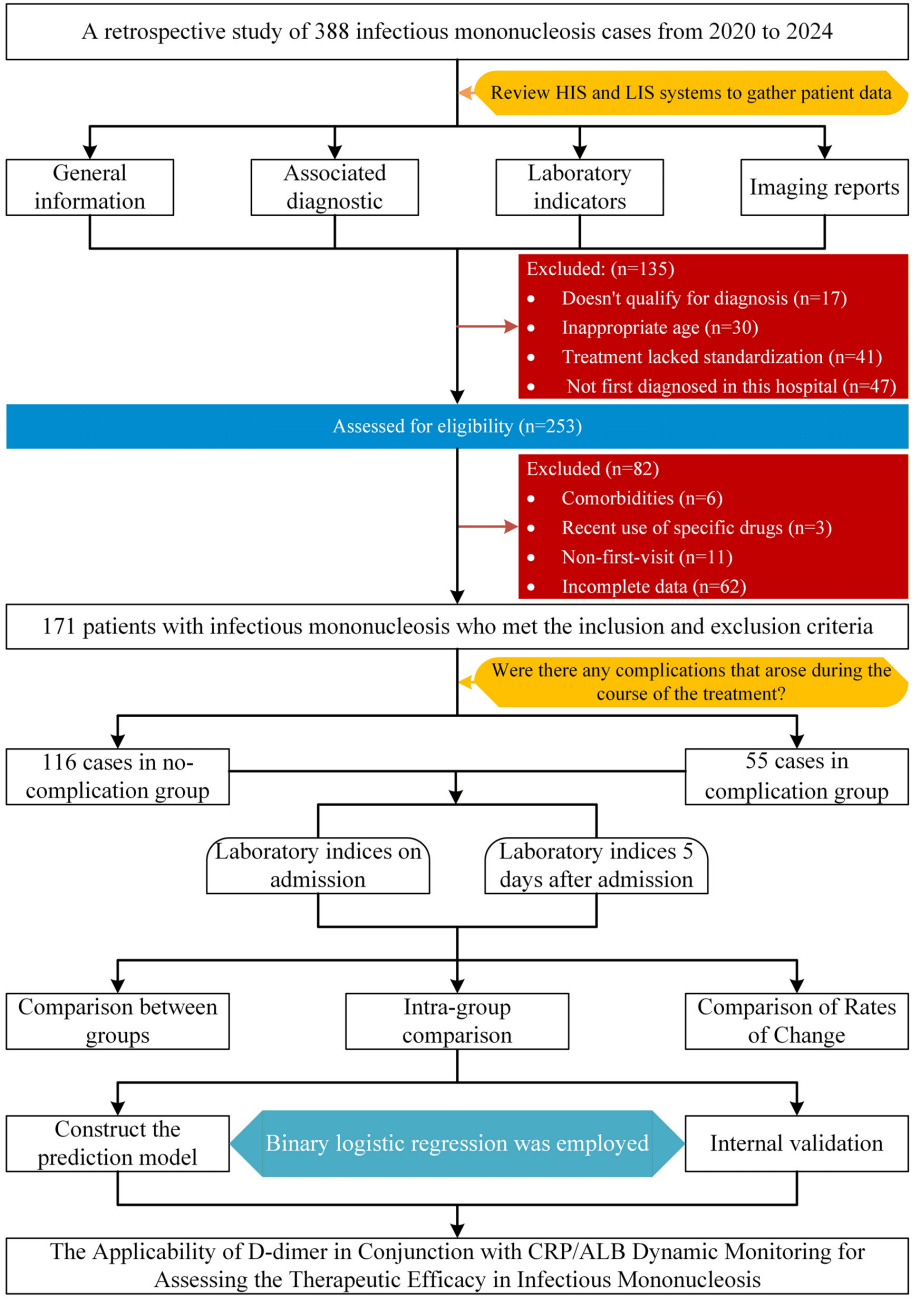
Study flow diagram.

The determination of the sample size was conducted utilizing the exact Clopper–Pearson method to estimate the complication rate (proportion) of infectious mononucleosis, with a two-sided 95% confidence interval. Drawing on a previous study involving 40 cases, which reported a complication rate of 30.0%, it was calculated that a minimum of 155 participants would be necessary to ensure that the width of the confidence interval does not exceed 0.15. These calculations were executed using PASS 2025 software (version 25.0.2). Utilizing the electronic medical record system, we retrospectively identified 388 patients who had been diagnosed with infectious mononucleosis and subsequently hospitalized for treatment at our institution between January 2020 and December 2024. Patients were initially categorized into two groups based on the presence or absence of complications: no-complication group and complication group. Following the application of predefined inclusion and exclusion criteria, a total of 171 patients were included in the final analysis, consisting of 116 patients without complications and 55 patients who developed complications. The event per variable (EPV) ratio was calculated to be 13.75 (55 events / 4 predictor variables = 13.75), surpassing the commonly recommended minimum threshold of 10 EPV, thereby ensuring the reliability of the logistic regression modeling. The detailed study flow is illustrated in Figure 1. This retrospective study was approved and exempted from written informed consent by the Ethical Review Committee of First People's Hospital of Linping District, Hangzhou (No. 2024023).

### 2.2 Inclusion and exclusion criteria

#### 2.2.1 Inclusion criteria

Patients were considered eligible for inclusion if they satisfied all of the following conditions: ① A diagnosis of infectious mononucleosis in accordance with the Expert Consensus on the Diagnosis and Treatment Principles of Epstein–Barr Virus Infection–Related Diseases in Children (12). ② An age range of 1 to 12 years. ③ Completion of a standardized full-course treatment regimen for infectious mononucleosis. ④ The initial diagnosis of infectious mononucleosis was made at our institution.

#### 2.2.2 Exclusion criteria

Patients were excluded from the study if they met any of the following conditions: ① presence of concomitant malignant tumors, autoimmune diseases, or hematologic disorders (*n* = 2); ② critical illness at onset accompanied by concurrent infections other than Epstein-Barr virus (EBV) (*n* = 3); ③ presence of psychiatric disorders or congenital malformations (*n* = 1); ④ treatment with immunosuppressive agents within 1 month prior to admission (*n* = 2); ⑤ recent use of medications known to affect coagulation function or related parameters (*n* = 1);⑥ non-first-visit status, defined as having been previously diagnosed or treated for the same condition either elsewhere or at our institution (*n* = 11);⑦ incomplete clinical data or missing relevant auxiliary examination results (*n* = 62).

### 2.3 Treatment protocol

Patients were administered standard treatment in accordance with the Expert Consensus on Epstein–Barr Virus Infection–Related Diseases in Children (12). The treatment protocol included the following components: (1) Supportive care, which involved maintaining fluid and electrolyte balance, ensuring adequate rest, and providing intravenous nutritional support when necessary. (2) Symptomatic treatment, which entailed the administration of ibuprofen or acetaminophen for antipyresis in cases where body temperature reached or exceeded 38.5 °C. (3) Management of complications, which included hepatoprotective therapy (e.g., reduced glutathione, and glycyrrhizic acid preparations) for patients with liver injury (13), glucocorticoid therapy (prednisolone 1–2 mg/kg/day) for those with upper airway obstruction, and pathogen-directed anti-infective therapy for patients with pneumonia, guided by etiological findings.

### 2.4 Laboratory assays

**Figure 2:**
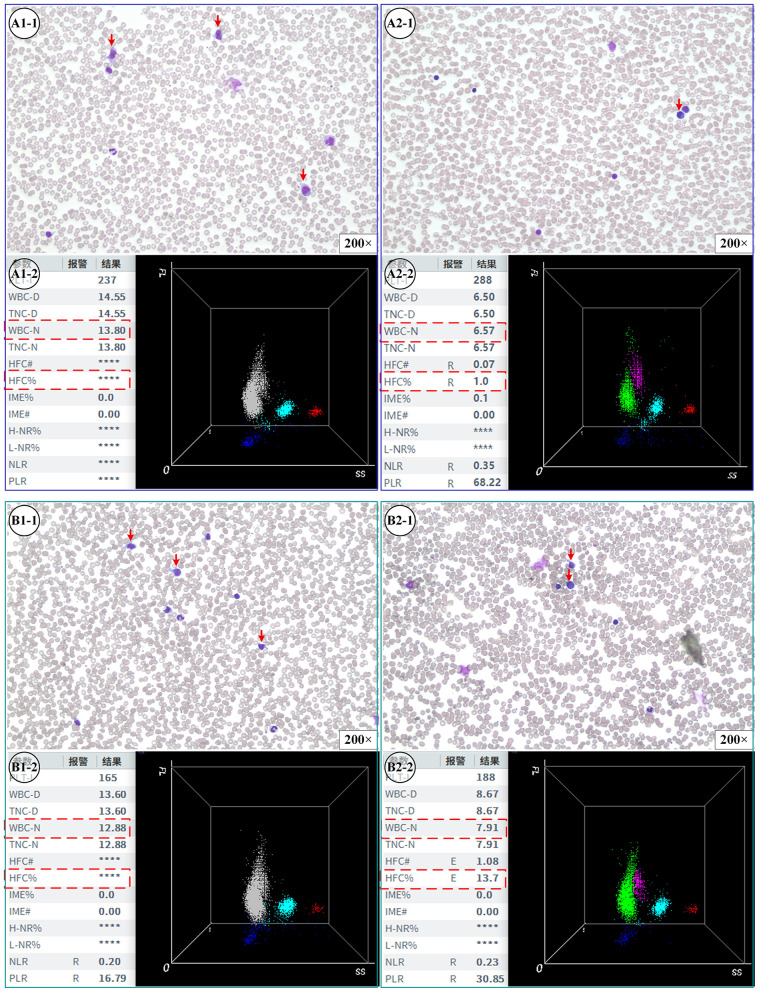
Cytomorphologic features and scattergram analysis of peripheral blood before and after treatment in children with IM. Representative peripheral blood smears (Wright-Giemsa stain, original magnification × 200) and corresponding three-dimensional scattergrams (Mindray BC-7500 hematology analyzer) are shown for two distinct clinical presentations. **(A)** Patient without complications: **(A1-1)** Admission: Peripheral blood smear demonstrating the presence of atypical lymphocytes (indicated by red arrows). **(A1-2)** Admission: Corresponding scattergram and hematological parameters. Note the cell clusters in the high-fluorescence region. **(A2-1, A2-2)** Post-treatment (Day 5): Follow-up microscopy showing normalized morphology **(A2-1)** and the corresponding post-treatment scattergram **(A2-2)**. **(B)** Patient with complications: **(B1-1)** Admission: Smear revealing atypical lymphocytes (red arrows). **(B1-2)** Admission: Scattergram data displaying distinct cell populations. **(B2-1, B2-2)** Post-treatment (Day 5): Comparative morphology **(B2-1)** and scattergram metrics **(B2-2)** following 5 days of therapy. Scattergram Key: Clusters differentiate cell populations based on fluorescence (FL), side scatter (SS), and forward scatter properties. The High-Fluorescence Cell (HFC) region typically correlates with the presence of atypical lymphocytes or blasts. H-NR% / L-NR%: High/Low nucleic acid fluorescence ratio; HFC# / HFC%: High-Fluorescence Cell count / percentage; IME% / IME#: Immature Eosinophils (or Immature Cells) percentage / count; NLR: Neutrophil-to-Lymphocyte Ratio; PLR: Platelet-to-Lymphocyte Ratio; TNC-D / TNC-N: Total Nucleated Cell count (Diff / WNB channel); WBC-D / WBC-N: White Blood Cell count (Diff / WNB channel).

Within 24 h of admission and again on the fifth day of treatment, D-dimer levels, complete blood counts, and biochemical parameters were assessed utilizing a Sysmex CS 5100 automated coagulation analyzer, a Mindray BC-7500 CS automated hematology analyzer, and a Beckman AU5800 automated biochemical analyzer, respectively. All procedures were conducted in strict accordance with the manufacturers' operational guidelines. The established reference range for D-dimer in adult patients is < 0.55 mg/L FEU. In accordance with the national standard WS/T 402-−2024, this reference range for adults was validated and confirmed to be applicable to pediatric populations at our institution (14). Consequently, a unified D-dimer reference interval of < 0.55 mg/L FEU was employed in this study. Concurrently, atypical lymphocytes percentage (ALC) were examined by two technicians using an Olympus CX43 microscope. When necessary, this evaluation was supplemented with high-fluorescence cell (HFC%) data provided by the Mindray BC-7500 CS automated hematology analyzer. Figure 2 presents representative atypical lymphocyte morphology and corresponding hematologic parameters from one patient in each group.

### 2.5 Imaging examinations and diagnosis

Radiological assessments were performed utilizing a Siemens Multix Fusion Max digital radiography system in conjunction with a SOMATOM Definition AS+ spiral CT scanner.

### 2.6 Observation indicators

(1) General Characteristics: The study evaluated patient demographics and clinical history, including age, sex, pre-existing medical conditions, and prior infections with Epstein–Barr virus (EBV) and cytomegalovirus (CMV). Additionally, the duration of fever and length of hospitalization were recorded.

(2) Laboratory Parameters: Within 24 h of admission and on the fifth day following treatment, a comprehensive blood analysis was conducted, including the assessment of white blood cell (WBC) count, absolute neutrophil count, absolute lymphocyte count, and the neutrophil-to-lymphocyte ratio (NLR), calculated as the absolute neutrophil count divided by the absolute lymphocyte count. Additionally, the percentage of atypical lymphocytes, D-dimer levels, C-reactive protein (CRP), and albumin concentrations were evaluated. Subsequently, several derived indices were calculated: ① the CRP/albumin ratio, determined by dividing CRP by albumin; ② the D-dimer change rate (ΔD-dimer), calculated as [D-dimer (pre-treatment)–D-dimer (post-treatment)] / D-dimer (pre-treatment) × 100%; ③ the WBC change rate (ΔWBC), determined as [WBC (pre-treatment) – WBC (post-treatment)] / WBC (pre-treatment) × 100%; ④ the absolute neutrophil count change rate (ΔN#), calculated as [absolute neutrophil count (pre-treatment)–absolute neutrophil count (post-treatment)] / absolute neutrophil count (pre-treatment) × 100%; ⑤ the absolute lymphocyte count change rate (ΔL#), determined as [absolute lymphocyte count (pre-treatment)–absolute lymphocyte count (post-treatment)] / absolute lymphocyte count (pre-treatment) × 100%; ⑥ Atypical lymphocyte percentage change rate (ΔALC) = [atypical lymphocyte percentage (pre-treatment)–atypical lymphocyte percentage (post-treatment)] / atypical lymphocyte percentage (pre-treatment) × 100%; ⑦ Albumin change rate (ΔALB) = [albumin (pre-treatment)–albumin (post-treatment)] / albumin (pre-treatment) × 100%; ⑧ CRP change rate (ΔCRP) = [CRP (pre-treatment)–CRP (post-treatment)] / CRP (pre-treatment) × 100%; ⑨ NLR change rate (ΔNLR) = [NLR (pre-treatment)–NLR (post-treatment)] / NLR (pre-treatment) × 100%; ⑩ CRP/albumin change rate (ΔCRP/ALB) = [CRP/albumin (pre-treatment)–CRP/albumin (post-treatment)] / CRP/albumin (pre-treatment) × 100%.

(3) The criteria for complications were defined as follows: ① **Liver injury** (25 cases): Defined as alanine aminotransferase (ALT) >3 × upper limit of normal (ULN) or total bilirubin >2 × ULN during the inpatient period **(****15****)**; ② **Upper airway obstruction (10 cases)**: Identification of grade III or higher tonsillar enlargement, severe pharyngeal edema, or cervical lymph nodes >3 cm, accompanied by dyspnea, dysphagia, or sleep apnea; ③ **Pneumoni**a (2 cases were mild): Diagnosed based on the *Guidelines for the Management of Community-Acquired Pneumonia in Children (2024 Edition)* (16), requiring ≥2 criteria: clinical symptoms (fever, cough, tachypnea), physical signs (rales, consolidation), or imaging evidence; ④ **Persistent high fever** (18 cases): Body temperature >39.5 °C with limited response to antipyretics, persisting for >5–7 days (17). In this study cohort, the clinical severity among the various complication subtypes was relatively uniform. All 55 cases with complications were classified as non-severe and were managed in the general ward; none necessitated admission to the intensive care unit (ICU) or required invasive mechanical ventilation. Notably, the two cases of pneumonia were mild and were effectively treated with oral antibiotics. In this study, complications such as liver injury, upper airway obstruction, pneumonia, and persistent high fever were combined into a single composite endpoint due to a limited sample size (*n* = 55) and their shared underlying causes of systemic hyperinflammation, immune activation, and endothelial/coagulation dysfunction in complicated IM. This approach aligns with our focus on systemic biomarkers like D-dimer and CRP/ALB.

### 2.7 Statistical analysis

Data analysis was conducted utilizing SPSS version 26.0 statistical software. Due to the study's retrospective design, we used complete case analysis, excluding patients with incomplete clinical or laboratory data as outlined in our exclusion criteria. Continuous data exhibiting a normal distribution are reported as mean ± standard deviation ($\bar{x}$ ± s), with intergroup comparisons assessed via the independent samples *t*-test. For continuous data not conforming to a normal distribution, values are expressed as median [interquartile range] [M (Q1, Q3)], and intergroup comparisons are executed using the Mann-Whitney U test. Categorical data are presented as frequencies (percentages), with intergroup comparisons performed using the chi-squared (χ^2^) test. Logistic regression analysis was applied to identify risk factors affecting treatment outcomes, employing stepwise variable selection through the Forward Likelihood Ratio (LR) method (entry criterion: *P* < 0.05; removal criterion: *P* > 0.10). The Box-Tidwell test confirmed the linear relationship between each continuous variable (days of fever, ΔALC, ΔD-dimer, and ΔCRP/ALB) and the log-odds of the outcome, as interaction terms with their natural logarithms were not significant (all *P* > 0.05), satisfying the linearity assumption. Receiver operating characteristic (ROC) curves were generated, and the area under the curve (AUC) was calculated to assess the predictive performance of each indicator. Statistical significance was determined at the *P* < 0.05 level. A random number table method was employed to allocate the 171 children diagnosed with IM into a training set (*n* = 119) and an internal validation set (*n* = 52) in a 7:3 ratio. Model validation was performed through bootstrap resampling (*n* = 1000) for internal validation, and the optimism-corrected C-index was calculated.

## 3 Results

### 3.1 General characteristics and clinical stratification of the two groups

A total of 171 enrolled children were included (116 in the no-complication group and 55 in the complication group). As shown in Table 1, there were no significant differences in baseline demographic characteristics, specifically age, sex, and History of allergic diseases (all *P* > 0.05). However, the complications group exhibited a more severe clinical course, manifesting as a significantly prolonged duration of fever (median: 3.0 vs. 2.0 days, *P* < 0.001) and total disease course (*P* = 0.037).

**Table 1:** General characteristics of study population.

Variables	Total	No-complication group	Complication group	Statistic	*P* value
Cases	171	116	55	—	—
Age (year), M (Q1, Q3)	5 (3, 7)	5 (3, 7)	5 (3.5, 6.5)	−0.177^*^	0.860
Gender, *n* (%)				1.731^#^	0.188
Male	84 (49.12)	61 (52.59)	23 (41.82)		
Female	87 (50.88)	55 (47.41)	32 (58.18)		
Fever (day), M (Q1, Q3)	3.0 (2.0, 4.0)	2.0 (1.0, 3.0)	3.0 (2.0, 5.0)	−3.687^*^	< 0.001
Course (day), M (Q1, Q3)	8.0 (6.0, 9.0)	7.0 (6.0, 9.0)	8.0 (6.5, 10.0)	−2.082^*^	0.037
History of allergic diseases, *n* (%)				0.001^#^	0.980
Yes	11 (6.43)	8 (6.90)	3 (5.45)		
No	160 (93.57)	108 (93.10)	52 (94.55)		
EB-virus infection, *n* (%)				0.066^#^	0.798
Yes	154 (90.06)	104 (89.66)	50 (90.91)		
No	17 (9.94)	12 (10.34)	5 (9.09)		
Cytomegalovirus Infection, *n* (%)				0.039^#^	0.843
Yes	10 (5.85)	6 (5.17)	4 (7.27)		
No	161 (94.15)	110 (94.83)	51 (92.73)		

^*^: Mann-Whitney test;

^#^: Chi-square test; M: Median; Q1: 1st Quartile; Q3: 3rd Quartile; History of allergic diseases included asthma, eczema, and food allergy.

### 3.2 Differences in laboratory parameters before and after treatment between the two groups

**Figure 3:**
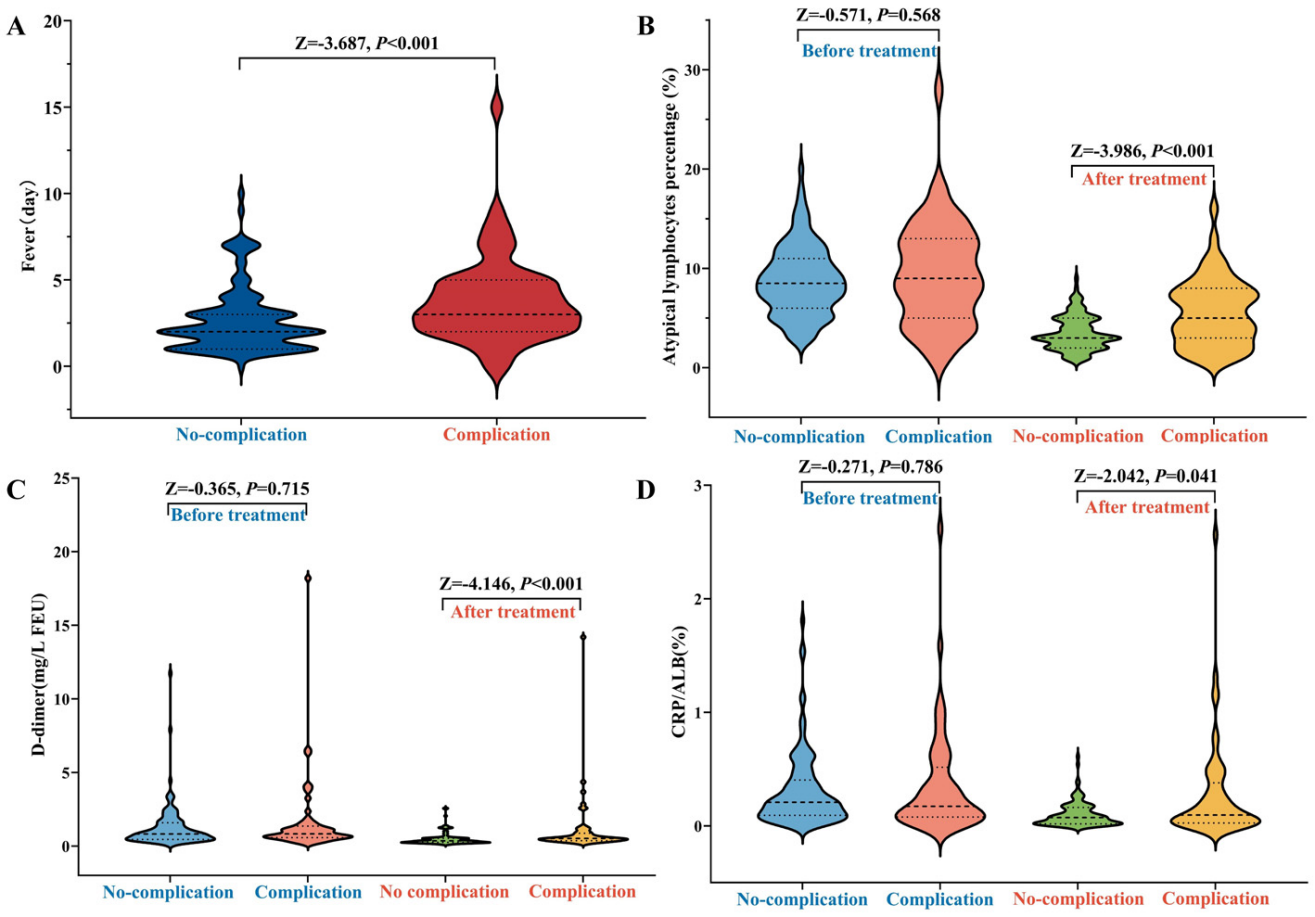
The level of clinical and laboratory parameters before and after treatment in children with IM. **(A)** Duration of fever; **(B)** Percentage of atypical lymphocytes; **(C)** D-dimer; **(D)** CRP/ALB ratio.

Baseline laboratory parameters obtained on admission—including WBC, neutrophils, lymphocytes, atypical lymphocyte percentage (ALC%), D-dimer, CRP, and the CRP/ALB ratio—were comparable between the two groups (all *P* > 0.05), indicating a balanced clinical profile at disease onset. However, significant divergence was observed after 5 days of treatment. Compared with the no-complications group, patients with complications retained significantly higher median levels of D-dimer (0.52 vs. 0.36 mg/L; *P* < 0.001), CRP (*P* = 0.044), and CRP/ALB ratio (*P* = 0.041), while albumin levels were lower (*P* = 0.044). Additionally, the resolution of atypical lymphocytosis was significantly less pronounced in the complications group (median ALC%: 5.00% vs. 3.00%; *P* < 0.001) (Table 2, Figure 3). Variations in these dynamic trajectories can be identified prior to the manifestation of overt clinical symptoms, thereby offering significant early warning potential. Specifically, a consistently elevated D-dimer level and CRP/ALB ratio, or a lack of significant decline within the initial 5 days, may suggest persistent coagulation activation and unresolved systemic inflammation. This observation could facilitate earlier risk stratification and warrant more vigilant monitoring.

**Table 2:** Comparison of laboratory indexes between the two groups before and after treatment.

Variables	Before treatment				After treatment			
	No-complication group	Complication group	Statistic	*P* value	No-complication group	Complication group	Statistic	*P* value
D-dimer (mg/L FEU), M (Q1, Q3)	0.82 (0.46, 1.57)	0.83 (0.58, 1.31)	−0.365^*^	0.715	0.36 (0.25, 0.53)	0.52 (0.39, 0.86)	−4.146^*^	< .001
WBC( × 10^9^/L), M (Q1, Q3)	12.50 (10.80, 15.43)	12.50 (10.45, 17.95)	−0.893^*^	0.372	7.95 (6.70, 10.83)	9.50 (7.30, 11.00)	−1.687^*^	0.092
NE# ( × 10^9^/L), M (Q1, Q3)	2.81 (1.98, 3.73)	2.83 (2.09, 4.04)	−0.954^*^	0.340	2.00 (1.42, 3.00)	2.04 (1.46, 2.72)	−0.410^*^	0.682
LY#( × 10^9^/L), Mean ± SD	8.71 ± 2.92	9.61 ± 3.90	1.528^#^	0.130	5.17 ± 2.06	6.32 ± 2.25	−3.299^*^	< .001
ALC (%), M (Q1, Q3)	8.5 (6.0, 11.0)	9.0 (5.5, 13.0)	−0.571^*^	0.568	3.0 (2.0, 5.0)	5.0 (3.0, 8.0)	−3.986^*^	< .001
ALB (g/L), Mean ± SD	39.71 ± 3.02	39.88 ± 3.34	0.330^#^	0.742	40.20 ± 3.06	39.23 ± 2.59	−2.026^#^	0.044
CRP (mg/L), M (Q1, Q3)	7.90 (3.98, 16.00)	7.40 (3.20, 17.55)	−0.279^*^	0.780	2.90 (0.70, 6.65)	3.90 (1.10, 13.05)	−2.019^*^	0.044
NLR, M (Q1, Q3)	0.34 (0.24, 0.42)	0.29 (0.21, 0.53)	−0.465^*^	0.642	0.43 (0.29, 0.60)	0.31 (0.24, 0.46)	−2.209^*^	0.027
CRP/ALB, M (Q1, Q3)	0.21 (0.10, 0.40)	0.17 (0.08, 0.48)	−0.271^*^	0.786	0.07 (0.02, 0.16)	0.10 (0.03, 0.35)	−2.042^*^	0.041

^*^: Mann-Whitney test,

^#^: *t* test; M: Median, Q1: 1st Quartile, Q3: 3st Quartile; WBC, White Blood Cell Count; NE#, Neutrophil Count; LY#, Lymphocyte Count; ALC, Atypical Lymphocyte percentage; ALB, Albumin; CRP, C-reactive protein; NLR, Neutrophil-to-Lymphocyte Ratio; CRP/ALB, C-reactive protein/Albumin.

### 3.3 Differences in dynamic change rates of main laboratory-related indicators between the two groups

**Figure 4:**
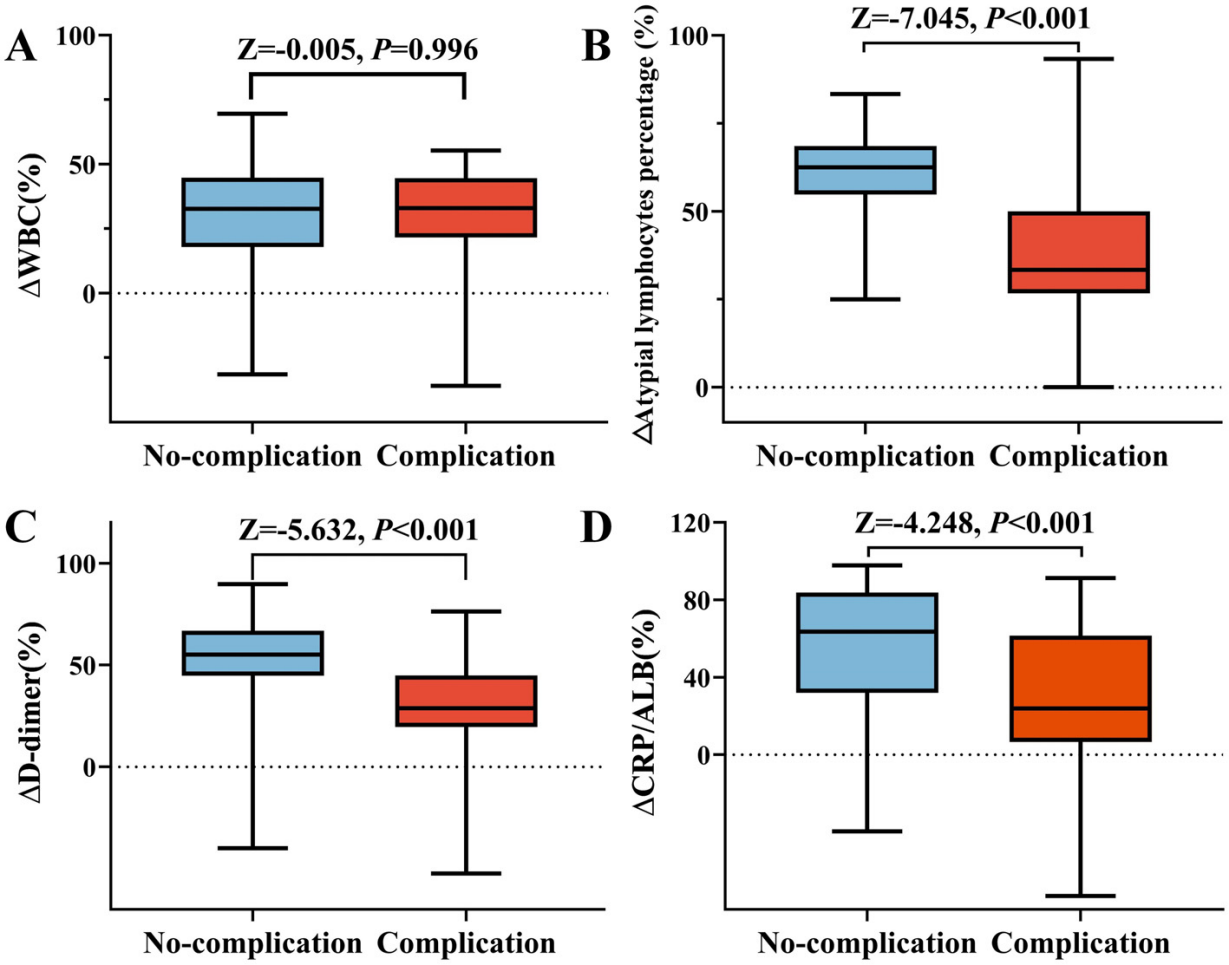
The change rate of primary laboratory parameters before and after treatment in children with IM. **(A)** WBC; **(B)** Percentage of atypical lymphocytes; **(C)** D-dimer; **(D)** CRP/ALB ratio.

Assessment of short-term changes (Δ) in laboratory parameters revealed distinct recovery patterns between the cohorts. Relative to the complications group, the no-complications group demonstrated significantly more pronounced reductions in inflammatory and coagulation markers, specifically ΔD-dimer (median: 55.10% vs. 28.77%; *P* < 0.001), ΔCRP (62.31% vs. 24.24%; *P* < 0.001), ΔALC (62.50% vs. 33.33%; *P* < 0.001), and the ΔCRP/ALB ratio (63.56% vs. 23.89%; *P* < 0.001). Of note, ΔALB followed a divergent trajectory: albumin levels trended toward normalization in the no-complications group (median change: −1.50%) whereas they continued to decline in the complications group (1.34%; *P* = 0.015). In contrast, change rates for white blood cell-related indices (ΔWBC, ΔNE#, ΔLY#, and ΔNLR) did not differ significantly between groups (all *P* > 0.05) and were subsequently excluded from predictive modeling (Table 3, Figure 4). The approximately two-fold differences in ΔD-dimer (55.10% compared to 28.77%) and ΔALC (62.50% compared to 33.33%) between the groups suggest distinct immunological pathways: rapid viral clearance in uncomplicated cases vs. sustained immune activation in complicated cases.

**Table 3:** Comparison of the rate of change in laboratory indices between the two groups before and after treatment.

Variables	Total	No-complication group	Complication group	Statistic	*P* value
Cases	171	116	55	—	—
ΔD-dimer (%), M (Q1, Q3)	50.00 (29.27, 63.56)	55.10 (46.03, 66.72)	28.77 (19.82, 44.41)	−5.632^*^	<0.001
ΔWBC (%), M (Q1, Q3)	32.98 (18.95, 44.38)	32.75 (18.04, 44.39)	32.98 (22.07, 44.21)	−0.005^*^	0.996
ΔNE# (%), M (Q1, Q3)	23.69 (−7.00, 51.69)	23.94 (−12.14, 49.99)	23.29 (−3.34, 53.43)	−0.217^*^	0.829
ΔLY# (%), M (Q1, Q3)	40.67 (18.75, 54.17)	42.58 (20.78, 56.89)	35.84 (13.14, 47.69)	−1.883^*^	0.060
ΔALB (%), Mean ± SD	−0.59 ± 7.12	−1.50 ± 7.54	1.34 ± 5.76	2.468^#^	0.015
ΔCRP (%), M (Q1, Q3)	52.63 (19.84, 76.19)	62.31 (32.92, 82.89)	24.24 (9.14, 56.46)	−4.068^*^	<0.001
ΔALC (%), M (Q1, Q3)	58.33 (40.00, 66.67)	62.50 (55.30, 67.31)	33.33 (26.67, 50.00)	−7.045^*^	<0.001
ΔNLR (%), M (Q1, Q3)	−18.46 (−97.62, 24.01)	−20.52 (−96.89, 18.13)	−11.25 (−96.93, 38.99)	−1.328^*^	0.184
ΔCRP/ALB (%), M (Q1, Q3)	52.32 (20.51, 76.38)	63.56 (35.38, 83.21)	23.89 (7.58, 57.91)	−4.248^*^	<0.001

^*^: Mann-Whitney test;

^#^: *t*-test; SD: standard deviation; M: Median, Q1: 1st Quartile, Q3: 3st Quartile; ΔD-dimer, Rate of D-dimer change; ΔWBC, Rate of White Blood Cell Count change; ΔNE#, Rate of Neutrophil Count change; ΔLY#, Rate of Lymphocyte Count change; ΔALC, Rate of Atypical Lymphocyte percentage change; ΔALB, Rate of Albumin change; ΔCRP, Rate of C-reactive protein change; ΔNLR, Rate of Neutrophil-to-Lymphocyte Ratio change; ΔCRP/ALB, Rate of C-reactive protein/Albumin change.

### 3.4 Analysis of factors influencing treatment response in IM

**Figure 5:**
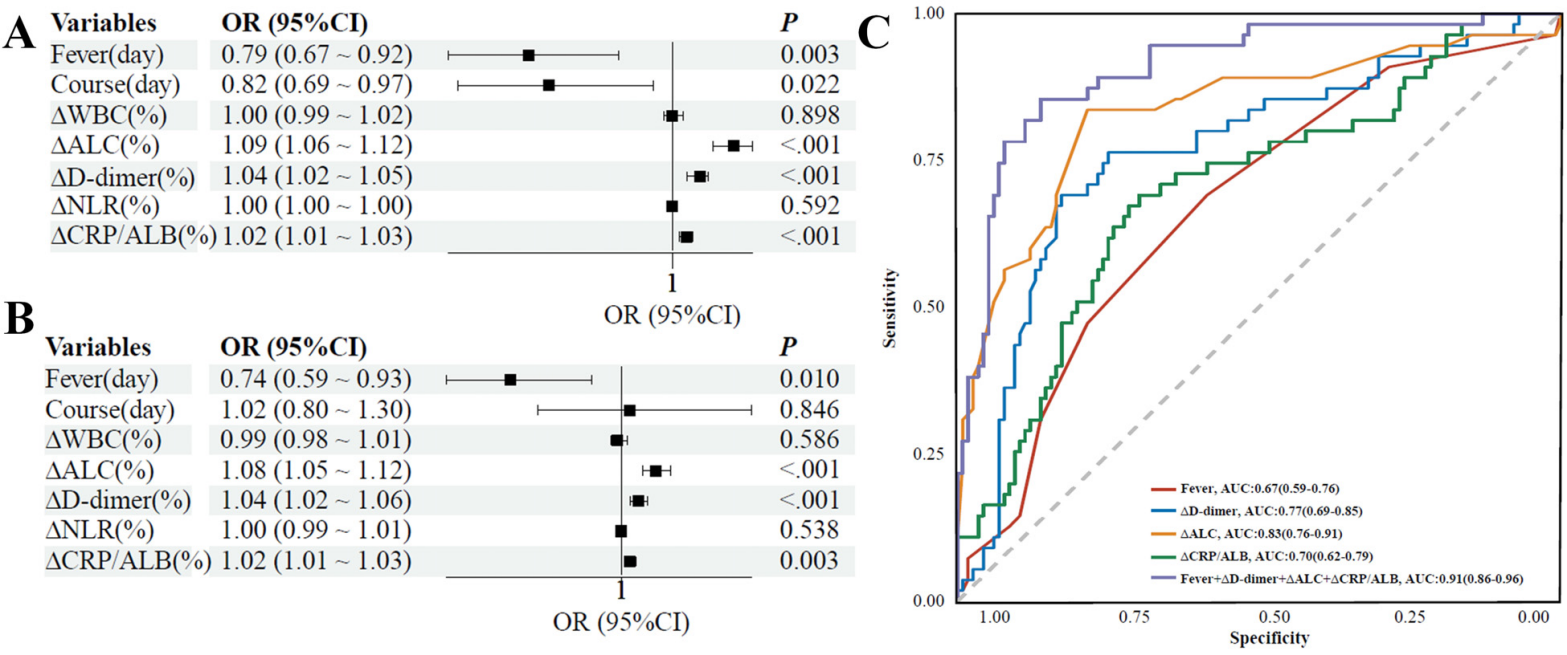
Forest plots and ROC curves of factors associated with treatment response in IM. **(A)** Univariate logistic regression analysis for identifying factors associated with treatment response. Odds Ratios (OR) and their 95% Confidence Intervals (CI) are shown. **(B)** Multivariate logistic regression analysis for identifying independent factors associated with treatment response. Odds Ratios (OR) and their 95% Confidence Intervals (CI) are shown. **(C)** Receiver Operating Characteristic (ROC) curves evaluating the predictive value of individual factors and their combination for treatment response. Each curve represents a factor or a combination of factors, with its corresponding Area Under the Curve (AUC) and 95% Confidence Interval displayed in the legend.

To identify independent determinants of a favorable prognosis (no-complication), binary logistic regression was performed. While fever duration, disease course, and the dynamic reduction rates (Δ) of ALC, D-dimer, and CRP/ALB were all identified as potential predictors in univariate analysis (Figure 5A), disease course lost statistical significance after multivariable adjustment. In the final adjusted model, four independent predictors emerged: shorter fever duration was significantly associated with a higher likelihood of recovery (OR = 0.74, 95% CI: 0.59–0.93; *P* = 0.010). Moreover, greater magnitude of reduction in biomarkers independently predicted favorable outcomes, including ΔALC (OR = 1.08, 1.05–1.12; *P* < 0.001), ΔD-dimer (OR = 1.04, 1.02–1.06; *P* < 0.001), and ΔCRP/ALB (OR = 1.02, 1.01–1.03; *P* = 0.003). Collectively, these data suggest that rapid biomarker clearance and prompt defervescence are independent indicators of a positive therapeutic response (Table 4, Figure 5B). A 10% larger drop in ΔALC boosts the chances of an uncomplicated course by 2.16 times, while a similar reduction in ΔD-dimer raises these odds by 1.48 times. Conversely, each additional day of fever lowers the likelihood of an uncomplicated outcome by 26% (OR = 0.74). These adjusted estimates provide clearer benchmarks for risk communication and assist clinicians in establishing personalized monitoring thresholds. Using the optimal cut-off from ROC analysis, patients with a predicted probability below the threshold (85.0% sensitivity, 86.0% specificity) can be considered low-risk, while those at or above are high-risk, offering an initial two-tier system for clinical triage.

**Table 4:** Univariate and multivariate logistic regression analyses of factors associated with infectious mononucleosis.

Variables	Univariate analysis					Multivariate analysis				
	β	S.E	Z	*P*	OR (95%CI)	β	S.E	Z	*P*	OR (95%CI)
Fever (day)	−0.24	0.08	−2.99	0.003	0.79 (0.67 ~ 0.92)	−0.30	0.12	−2.59	0.010	0.74 (0.59 ~ 0.93)
Course (day)	−0.20	0.09	−2.28	0.022	0.82 (0.69 ~ 0.97)	0.02	0.12	0.19	0.846	1.02 (0.80 ~ 1.30)
ΔWBC (%)	0.01	0.01	0.13	0.898	1.00 (0.99 ~ 1.02)	−0.01	0.01	−0.54	0.586	0.99 (0.98 ~ 1.01)
ΔALC (%)	0.08	0.01	6.26	<0.001	1.09 (1.06 ~ 1.12)	0.08	0.02	5.18	<0.001	1.08 (1.05 ~ 1.12)
ΔD-dimer (%)	0.04	0.01	4.60	<0.001	1.04 (1.02 ~ 1.05)	0.04	0.01	3.92	<0.001	1.04 (1.02 ~ 1.06)
ΔNLR (%)	−0.01	0.01	−0.54	0.592	1.00 (1.00 ~ 1.00)	−0.01	0.01	−0.62	0.538	1.00 (0.99 ~ 1.01)
ΔCRP/ALB (%)	0.02	0.01	4.19	<0.001	1.02 (1.01 ~ 1.03)	0.02	0.01	3.01	0.003	1.02 (1.01~ 1.03)

OR, Odds Ratio; CI, Confidence Interval; ΔWBC, Rate of White Blood Cell Count change; ΔALC, Rate of Atypical Lymphocyte percentage change; ΔD-dimer, Rate of D-dimer change; ΔNLR, Rate of Neutrophil-to-Lymphocyte Ratio change; ΔCRP/ALB, Rate of C-reactive protein/Albumin change.

### 3.5 Diagnostic analysis was performed using ROC curve analysis

To quantify the prognostic accuracy of the identified biomarkers, ROC curve analysis was performed. Of the individual predictors, ΔALC exhibited the strongest predictive capability, yielding an AUC of 0.83 (95% CI: 0.76–0.91). Other markers showed moderate accuracy, including ΔD-dimer (AUC: 0.77; 95% CI: 0.69–0.85), ΔCRP/ALB (AUC: 0.70; 95% CI: 0.62–0.79), and fever duration (AUC: 0.67; 95% CI: 0.59–0.76). Notably, the composite model—incorporating all four independent factors—offered superior discrimination compared with any single marker. This integrated approach achieved an AUC of 0.91 (95% CI: 0.86–0.96), with a sensitivity of 85.0%, specificity of 86.0%, and overall accuracy of 86.0%, suggesting potential clinical utility (Figure 5C, Supplement Table 1). Importantly, the odds ratio derived from logistic regression quantifies the strength of association after controlling for other variables, while the area under the receiver operating characteristic curve (AUC) assesses the predictive accuracy of a variable in distinguishing between individual cases. Consequently, a variable may demonstrate a statistically significant independent association, as indicated by a significant odds ratio, yet still result in a relatively modest AUC if there is considerable overlap in the distributions between the groups being compared. From a clinical standpoint, ΔALC showed the best performance among markers, with 84.0% sensitivity and 91.0% NPV, indicating that patients with a high ΔALC response are likely free of complications. The composite model enhanced this, reaching a 95.0% NPV in validation, suggesting its potential as a “rule-out” tool to identify low-risk patients who may not need extra monitoring or extended hospital stays.

### 3.6 Validation of the predictive model for treatment response in IM

**Figure 6:**
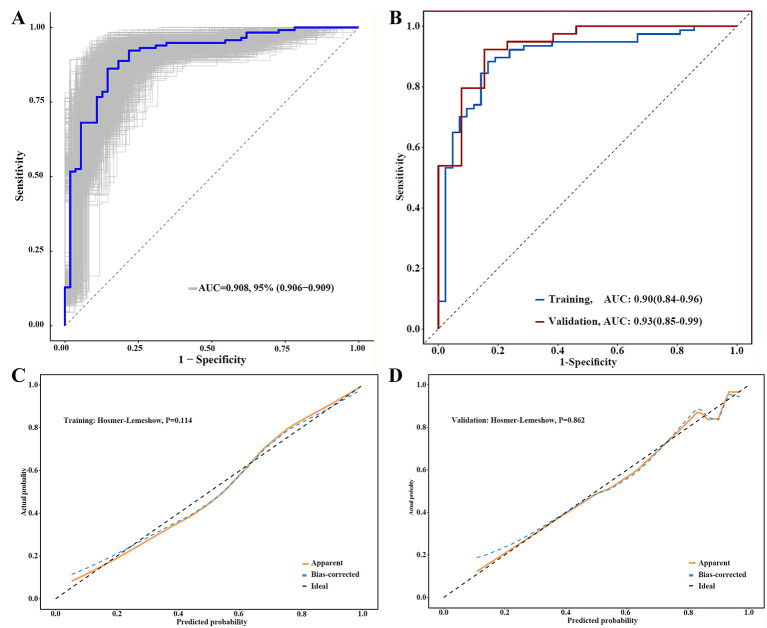
Validation of the prediction model for treatment response in IM. **(A)** Bootstrapped ROC curve (1000 resamples); **(B)** ROC curves for the training and validation cohorts; **(C)** Calibration curve in the training cohort; **(D)** Calibration curve in the validation cohort.

To evaluate the generalizability and robustness of the composite model, internal validation was performed. First, bootstrap validation with 1,000 resamples demonstrated highly stable predictive performance, yielding an AUC of 0.908 (95% CI: 0.906–0.909) (Figure 6A). In terms of discrimination, the model demonstrated favorable predictive accuracy in the training cohort, with an AUC of 0.90 (95% CI: 0.84–0.96). Crucially, this high discriminatory power was preserved in the validation cohort (AUC = 0.93, 95% CI: 0.85–0.99), suggesting minimal overfitting (Figure 6B). Regarding calibration, the Hosmer–Lemeshow test indicated good goodness-of-fit in both the training (*P* = 0.114) and validation cohorts (*P* = 0.862), revealing no significant deviation between predicted and observed risks. Corroborating these findings, the calibration plots showed high concordance, with bias-corrected curves closely approximating the ideal 45-degree diagonal across the probability range (Figures 6C, D). Collectively, these metrics support the model's internal reliability and highlight its potential utility for future clinical decision-making.

## 4 Discussion

In this study, we developed and validated a multiparametric dynamic evaluation model designed to assess the therapeutic risk of complications in pediatric patients with infectious mononucleosis (IM) according to their therapeutic response. Within our cohort of 171 patients, the incidence of treatment non-response and associated complications was 32.2%. This figure is considerably lower than the 70.4% complication rate reported in a recent nationwide retrospective study encompassing over 24,000 hospitalized children in China (18). Such a discrepancy likely stems from inter-institutional heterogeneity; whereas the nationwide dataset reflects varying levels of care, our single-center cohort was managed under a strict protocol aligned with the 2021 Chinese expert consensus (12), which may have mitigated disease progression. Crucially, our composite model—incorporating fever duration and the dynamic kinetics of atypical lymphocytes percentage (ΔALC), D-dimer (ΔD-dimer), and the CRP/albumin ratio (ΔCRP/ALB)—demonstrated robust discriminatory power (AUC 0.91). By reflecting the interplay between immune dysregulation, coagulation activation, and the inflammation–nutrition axis, this tool enables the early identification of patients at high risk for complications, complementing the predictive capacity of standard clinical assessments (19).

A pivotal strength of this study lies in its shift from static baseline measurements to longitudinal kinetics, providing a refined perspective on disease evolution. While baseline parameters—such as an atypical lymphocyte fraction exceeding 10%—are established risk factors for complications like hepatic injury (OR 4.573) (20), these “static snapshots” fail to capture the host's dynamic capacity for immune resolution. In our cohort, the rate of decline in atypical lymphocytes (ΔALC) proved to be a superior predictor, underscoring that the trajectory of recovery reflects the underlying immunopathology more accurately than the initial magnitude of the response. This observation aligns with mechanistic evidence suggesting that persistent T-cell activation, rather than peak activation levels, is the primary driver of poor viral control and tissue damage (7). Similarly, the prognostic utility of the inflammation–nutrition axis appears to be context-dependent. Although baseline CRP/ALB ratios have demonstrated high predictive accuracy in acute, hyperinflammatory states such as severe COVID-19 (AUC 0.866) (10), their independent performance in our IM cohort was more modest (AUC 0.70). This discrepancy likely stems from the distinct inflammatory profile of IM, which is characterized by a protracted cytokine response rather than the rapid, fulminant “storm” seen in critical illnesses. Consequently, monitoring the rate of change (ΔCRP/ALB), rather than a singular absolute value, serves as a more sensitive indicator of the cumulative physiological stress imposed on the inflammation–nutrition axis.

The integration of dynamic D-dimer kinetics (ΔD-dimer) significantly augments the predictive robustness of our model (OR 1.04), extending the prognostic landscape beyond traditional static measurements. While absolute D-dimer elevations are established biomarkers of severity in pediatric infectious syndromes—evidenced by their utility in severe *Mycoplasma pneumoniae* pneumonia (AUC 0.721) (21) and in distinguishing IM from the life-threatening Epstein-Barr virus-associated hemophagocytic lymphohistiocytosis (EBV-HLH) (AUC 0.869) (22)—these studies have predominantly relied on single time-point assessments. Our findings refine this paradigm by demonstrating that the rate of change within the early therapeutic window may offer improved prognostic value. Mechanistically, this longitudinal trajectory likely serves as a real-time surrogate for the intensity of immunothrombosis and endothelial activation, which are central drivers of IM-associated complications (19). Unlike a static baseline value, which quantifies the initial insult, a failure of D-dimer to decline (or a paradoxical rise) signals persistent coagulation activation driven by unresolved macrophage activity. Consequently, ΔD-dimer provides a more nuanced insight into the disease trajectory, facilitating the preemptive identification of patients at risk for systemic deterioration. This dynamic assessment is particularly valuable because subtle laboratory changes frequently precede the subjective findings observed during a physical examination. Although direct cytokine profiling was not feasible within the scope of this study due to the self-limiting nature of infectious mononucleosis (IM) and the limited number of eligible hospitalized patients for longitudinal cytokine assessment, existing literature suggests that IM is characterized by a prolonged inflammatory response rather than an acute cytokine storm. This is evidenced by the sustained elevation of cytokines such as IL-18 and IL-10, which are associated with disease severity, endothelial damage, and immune thrombosis (23, 24). This immunological context further underscores the relevance of utilizing markers that reflect inflammation and coagulation, such as changes in D-dimer (ΔD-dimer) and the ratio of C-reactive protein to albumin (ΔCRP/ALB), for evaluating the progression of IM.

The translational significance of this model lies in its potential to inform risk-stratified management strategies. By providing precise and early risk delineation, the model has the potential to facilitate actionable and tiered clinical responses. For patients identified as low-risk (with predicted probabilities below the threshold, characterized by prompt resolution of fever and rapid clearance of biomarkers), clinicians may consider transitioning to oral symptomatic management and planning for discharge by day five, with follow-up reserved for routine outpatient review. Conversely, for high-risk patients (those at or above the threshold, exhibiting delayed clearance of D-dimer or persistently elevated CRP/ALB levels), a significantly different approach is recommended. This includes extending inpatient observation, conducting targeted assessments for subclinical organ involvement (such as monitoring hepatic enzymes and performing abdominal ultrasonography), and lowering the threshold for initiating glucocorticoid therapy before overt clinical deterioration occurs. Crucially, unlike mechanistic studies that rely on high-dimensional, resource-intensive technologies like single-cell RNA sequencing, our algorithm exclusively leverages ubiquitous, cost-effective laboratory parameters. This pragmatic design suggests high scalability and feasibility for future implementation even in resource-limited settings. By integrating dynamic patient kinetics rather than relying on static snapshots, the model addresses a critical unmet need, bridging the gap between complex pathophysiological insights and accessible, individualized bedside care.

By the fifth day, clinical indicators such as defervescence frequently offer immediate bedside evidence of patient improvement. Nevertheless, relying solely on clinical observation can be subjective and may overlook subclinical persistence of inflammation or coagulopathy. The principal advantage of our model lies not merely in traditional “prediction,” but in providing an objective adjunct metric to monitor the disease trajectory. By quantifying the resolution of the inflammation-coagulation-nutrition axis, for instance through changes in D-dimer and CRP/ALB ratios, this tool offers a standardized metric to ensure that clinical improvement is corroborated by physiological recovery. This approach helps prevent premature discharge and identifies “non-responders” who may require intensified care, even when clinical signs are ambiguous.

Several limitations warrant careful consideration. Firstly, the retrospective, single-center design inherently introduces selection bias and potential unmeasured confounding factors. Additionally, the confinement of the study population to hospitalized children introduces spectrum bias, as patients treated in outpatient settings or those with milder disease courses were systematically excluded. This exclusion may have led to an inflated observed complication rate of 32.2% compared to the broader infectious mononucleosis (IM) population, thus limiting the generalizability of our model to unselected, community-based populations. Consequently, external validation through large-scale, multicenter prospective cohorts is essential to confirm the model's applicability across diverse clinical settings. Second, although the two groups were balanced at baseline regarding age, sex, and allergic history (all *P* > 0.05), the complication group demonstrated a significantly longer duration of fever at admission (median 3.0 vs. 2.0 days, *P* < 0.001). This finding suggests residual differences in disease severity at admission that may not have been fully accounted for by the multivariable model. Additionally, the timing of therapy initiation, particularly concerning the administration of glucocorticoids or hepatoprotective agents, was not standardized as a covariate in the regression analysis. This represents a potential source of unmeasured confounding that could have affected the trajectories of biomarkers by day 5. To address these confounders, future prospective studies should incorporate predefined severity scoring systems and protocol-driven criteria for treatment initiation. Third, owing to institutional resource limitations during the study period (2020–2024), the majority of patients were not subjected to EBV-DNA testing, which impeded a thorough analysis of viral kinetics. Future multicenter prospective studies that incorporate standardized EBV-DNA monitoring protocols could improve predictive accuracy when integrated with our biochemical biomarkers. Fourth, although our model utilizes readily accessible macroscopic biomarkers, it lacks the detailed pathophysiological insights provided by advanced immunophenotyping. Future research should focus on translating pertinent transcriptomic signatures into simplified clinical panels to augment the capabilities of our model. Finally, the follow-up was limited to the acute hospitalization phase, assessing biomarkers only at baseline and day 5. This short-term evaluation might miss intermediate peaks or complex fluctuations. Thus, longitudinal studies with frequent post-baseline assessments beyond discharge are needed to fully map biomarker trajectories and predict long-term outcomes like chronic active EBV infection (CAEBV). Additionally, we recognize that stepwise regression for variable selection may lead to biased *P*-values and overfitting. Nonetheless, our bootstrap validation with 1,000 resamples (Figure 6A) helps mitigate these risks by confirming stable predictive performance.

## 5 Conclusion

This study suggests that early short-term changes (from baseline to day 5) in systemic inflammation markers (CRP/ALB), coagulation indicators (D-dimer), atypical lymphocyte percentage (ALC), and fever duration may serve as preliminary yet promising predictors of complication risk in pediatric infectious mononucleosis. By leveraging widely accessible routine biomarkers, this multidimensional approach may provide clinicians with a practical adjunct tool for bedside risk stratification. The composite model demonstrated promising internal discriminatory performance (training AUC = 0.90; validation AUC = 0.93, NPV = 93.0%), indicating its potential utility as a “rule-out” instrument for excluding complications in patients at low risk. Clinically, rapid biomarker resolution may support earlier discharge decisions when clinical stability is concurrently confirmed, whereas delayed clearance may suggest the need for intensified monitoring or timely therapeutic adjustments.

These findings should be interpreted with caution due to the study's retrospective, single-center design and relatively modest sample size, which may introduce selection bias and limit generalizability. The composite outcome, though based on shared hyperinflammatory mechanisms, includes varied clinical entities that require future subgroup analyses. While the model shows promise, it needs further external validation before clinical use. Large-scale, multicenter prospective studies with frequent longitudinal assessments extending beyond discharge are necessary to externally validate these short-term trajectories, compare the proposed model with existing clinical strategies, refine clinically actionable thresholds, and determine its optimal integration into standardized pediatric IM management. Future models should incorporate viral load kinetics and advanced immunological profiling to further enhance predictive accuracy.

## Data Availability

The original contributions presented in the study are included in the article/Supplementary material, further inquiries can be directed to the corresponding authors.
