# Supplementary material for: The clinical utility of combining D-dimer with the C-reactive protein/albumin ratio for assessing treatment response in pediatric infectious mononucleosis
Source: Front Med (Lausanne). 2026 May 15;13:1830883. doi: 10.3389/fmed.2026.1830883 (PMC13219324; doi:10.3389/fmed.2026.1830883)
Supplement: Supplementary file 1 [file Supplementary_Table_1.docx]

| **Supplement Table 1** Diagnostic Value of Laboratory Indicators for Predicting Treatment Outcomes in Infectious Mononucleosis Patients | | | | | | | | |
| --- | --- | --- | --- | --- | --- | --- | --- | --- |
| **Item** | **AUC (95%CI)** | **Accuracy (95%CI)** | **Sensitivity (95%CI)** | **Specificity (95%CI)** | **PPV (95%CI)** | **NPV (95%CI)** | **LR+** | **LR−** |
| Fever (day) | 0.67 (0.59-0.76) | 0.38 (0.31-0.46) | 0.31 (0.19-0.43) | 0.41 (0.32-0.50) | 0.20 (0.11-0.29) | 0.56 (0.45-0.66) | 0.53 | 1.68 |
| Laboratory indices |  |  |  |  |  |  |  |  |
| ∆D-dimer (%) | 0.77 (0.69-0.85) | 0.78 (0.71-0.84) | 0.69 (0.57-0.81) | 0.83 (0.76-0.90) | 0.66 (0.53-0.78) | 0.85 (0.78-0.92) | 4.06 | 0.37 |
| ∆ALC (%) | 0.83 (0.76-0.91) | 0.80 (0.73-0.86) | 0.84 (0.74-0.93) | 0.78 (0.71-0.86) | 0.65 (0.54-0.76) | 0.91 (0.85-0.97) | 3.82 | 0.21 |
| ∆CRP/ALB (%) | 0.70 (0.62-0.79) | 0.70 (0.62-0.76) | 0.69 (0.57-0.81) | 0.70 (0.61-0.78) | 0.52 (0.41-0.64) | 0.83 (0.75-0.90) | 2.30 | 0.44 |
| Fever + ΔD-dimer + ΔALC + ΔCRP/ALB | 0.91 (0.86-0.96) | 0.86 (0.80-0.91) | 0.85 (0.76-0.95) | 0.86 (0.80-0.92) | 0.75 (0.64-0.85) | 0.93 (0.88-0.98) | 6.07 | 0.17 |
| Cohort |  |  |  |  |  |  |  |  |
| Training | 0.90(0.84-0.96) | 0.86(0.78-0.92) | 0.83(0.72-0.95) | 0.87(0.80-0.95) | 0.78(0.66-0.90) | 0.91(0.84-0.97) | 6.38 | 0.20 |
| Validation | 0.93(0.85-0.99) | 0.89(0.77-0.96) | 0.85(0.650-0.99) | 0.90(0.80-0.99) | 0.73(0.50-0.96) | 0.95(0.87-0.99) | 8.50 | 0.17 |
| Note: AUC: Area Under Curve; CI: Confidence Interval; PPV=positive predictive value; NPV=negative predictive value; Abbreviation: ∆D-dimer: Rate of D-dimer change; ∆ALC: Rate of Atypical Lymphocyte percentage change; ∆CRP/ALB: Rate of C-reactive protein/Albumin change; LR+: positive likelihood ratio; LR−: negative likelihood ratio. | | | | | | | | |
